# *N*′-(2-Hy­droxy-4-meth­oxy­benzyl­idene)-3-nitro­benzohydrazide

**DOI:** 10.1107/S160053681104178X

**Published:** 2011-10-12

**Authors:** Chun-Bao Tang

**Affiliations:** a Department of Chemistry, Jiaying University, Meizhou 514015, People’s Republic of China

## Abstract

In the mol­ecule of the title compound, C_15_H_13_N_3_O_3_, an intra­molecular O—H⋯N hydrogen bond influences the planarity of the conformation; the dihedral angle between the benzene rings is 11.4 (3)°. In the crystal, mol­ecules are linked by N—H⋯O hydrogen bonds into chains in [101].

## Related literature

For general background to hydrazones, see: Rasras *et al.* (2010 ▶); Pyta *et al.* (2010 ▶); Angelusiu *et al.* (2010 ▶). For related structures, see: Fun *et al.* (2008 ▶); Singh & Singh (2010 ▶); Ahmad *et al.* (2010 ▶); Tang (2010 ▶, 2011 ▶). For hydrogen-bond motifs, see: Bernstein *et al.* (1995 ▶).
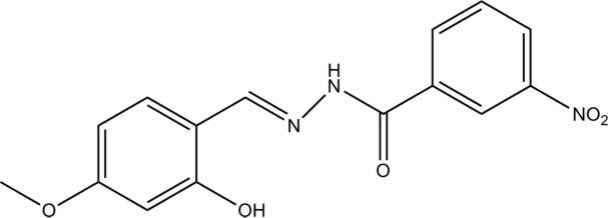


## Experimental

### 

#### Crystal data

C_15_H_13_N_3_O_5_
*M* *_r_* = 315.28
Monoclinic, 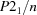

*a* = 6.0099 (12) Å
*b* = 33.575 (3) Å
*c* = 7.319 (2) Å
β = 94.235 (2)°
*V* = 1472.9 (5) Å^3^
*Z* = 4
Mo *K*α radiation
μ = 0.11 mm^−1^
*T* = 298 K
0.28 × 0.23 × 0.22 mm

#### Data collection

Bruker SMART CCD area-detector diffractometer
Absorption correction: multi-scan (*SADABS*; Sheldrick, 1996 ▶) *T*_min_ = 0.970, *T*_max_ = 0.976
7720 measured reflections
3155 independent reflections
1786 reflections with *I* > 2σ(*I*)
*R*_int_ = 0.032

#### Refinement

*R*[*F* ^2^ > 2σ(*F* ^2^)] = 0.056
*wR*(*F* ^2^) = 0.140
*S* = 1.02
3155 reflections
214 parameters
1 restraint
H atoms treated by a mixture of independent and constrained refinement
Δρ_max_ = 0.14 e Å^−3^
Δρ_min_ = −0.19 e Å^−3^

### 

Data collection: *SMART* (Bruker, 2002 ▶); cell refinement: *SAINT* (Bruker, 2002 ▶); data reduction: *SAINT*; program(s) used to solve structure: *SHELXS97* (Sheldrick, 2008 ▶); program(s) used to refine structure: *SHELXL97* (Sheldrick, 2008 ▶); molecular graphics: *SHELXTL* (Sheldrick, 2008 ▶); software used to prepare material for publication: *SHELXL97*.

## Supplementary Material

Crystal structure: contains datablock(s) global, I. DOI: 10.1107/S160053681104178X/cv5171sup1.cif

Structure factors: contains datablock(s) I. DOI: 10.1107/S160053681104178X/cv5171Isup2.hkl

Supplementary material file. DOI: 10.1107/S160053681104178X/cv5171Isup3.cml

Additional supplementary materials:  crystallographic information; 3D view; checkCIF report

## Figures and Tables

**Table 1:** Hydrogen-bond geometry (Å, °)

*D*—H⋯*A*	*D*—H	H⋯*A*	*D*⋯*A*	*D*—H⋯*A*
O1—H1⋯N1	0.82	1.90	2.618 (2)	146
N2—H2⋯O3^i^	0.90 (1)	1.93 (1)	2.806 (2)	165 (2)

Symmetry code: (i) 
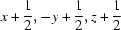
.

**Fig. 1.**
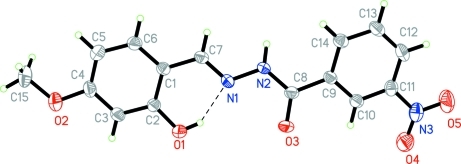
The molecular structure of (I) showing the atom-numbering scheme. Displacement ellipsoids are drawn at the 30% probability level. Hydrogen atoms are shown as spheres of arbitrary radius and hydrogen bond is drawn as a dashed line.

**Fig. 2.**
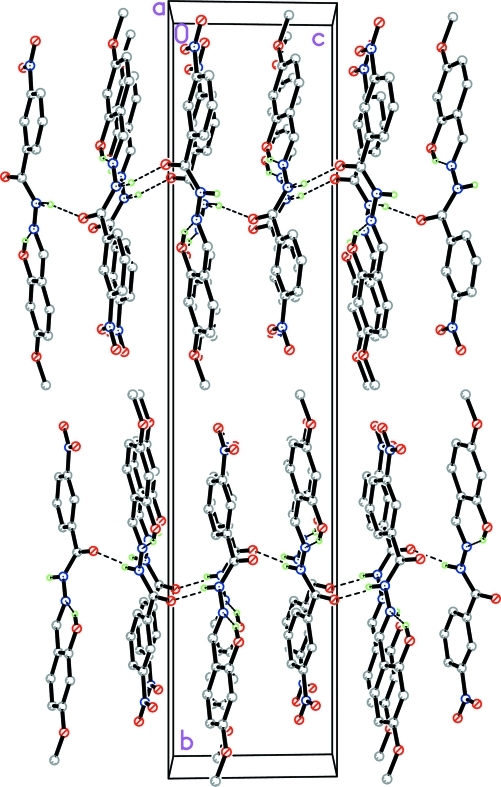
A portion of the crystal packing showing hydrogen bonds as dashed lines. H atoms non-involved in hydrogen bonding omitted for clarity.

## supplementary crystallographic information

### Comment

Hydrazone compounds have received much attention in biological and structural chemistry in the last years (Rasras *et al.*, 2010; Pyta *et al.*, 2010; Angelusiu *et al.*, 2010). Herewith we report the crystal structure of the title new hydrazone compound (I).

In (I) (Fig. 1), the bond lengths and angles are normal and comparable to those observed in the similar compounds (Fun *et al.*, 2008; Singh & Singh, 2010; Ahmad *et al.*, 2010; Tang, 2010, 2011). Intramolecular O1—H1···N1 hydrogen bond generates a S(6) ring motif (Bernstein *et al.*, 1995). The dihedral angle between the two benzene rings in the molecule is 11.4 (3)°.

In the crystal structure, the molecules are linked through intermolecular N—H···O hydrogen bonds (Table 1) into chains in [101] (Fig. 2).

### Experimental

2-Hydroxy-4-methoxybenzaldehyde (0.1 mmol, 15.2 mg) and 3-nitrobenzohydrazide (0.1 mmol, 18.1 mg) were dissolved in methanol (20 ml). The mixture was stirred at reflux for 10 min to give a clear yellow solution. Yellow crystals of the compound were formed by slow evaporation of the solvent over several days.

### Refinement

The amino H atom was located in a difference Fourier map and refined with the N—H distance restrained to 0.90 (1) Å and *U*_iso_(H) fixed to 0.081 Å^2^. Other H atoms were constrained to ideal geometries and refined as riding, with Csp^2^—H = 0.93 Å, C(methyl)—H = 0.96 Å, and O—H = 0.82 Å; *U*_iso_(H) = 1.2*U*_eq_(C) and 1.5*U*_eq_(O and C_methyl_).

### Crystal data

**Table:**

C_15_H_13_N_3_O_5_	*F*(000) = 656
*M**_r_* = 315.28	*D*_x_ = 1.422 Mg m^−^^3^
Monoclinic, *P*2_1_/*n*	Mo *K*α radiation, λ = 0.71073 Å
*a* = 6.0099 (12) Å	Cell parameters from 1287 reflections
*b* = 33.575 (3) Å	θ = 2.4–24.5°
*c* = 7.319 (2) Å	µ = 0.11 mm^−^^1^
β = 94.235 (2)°	*T* = 298 K
*V* = 1472.9 (5) Å^3^	Prism, yellow
*Z* = 4	0.28 × 0.23 × 0.22 mm

### Data collection

**Table:**

Bruker SMART CCD area-detector diffractometer	3155 independent reflections
Radiation source: fine-focus sealed tube	1786 reflections with *I* > 2σ(*I*)
graphite	*R*_int_ = 0.032
ω scans	θ_max_ = 27.0°, θ_min_ = 2.4°
Absorption correction: multi-scan (*SADABS*; Sheldrick, 1996)	*h* = −7→5
*T*_min_ = 0.970, *T*_max_ = 0.976	*k* = −42→32
7720 measured reflections	*l* = −9→9

### Refinement

**Table:**

Refinement on *F*^2^	Primary atom site location: structure-invariant direct methods
Least-squares matrix: full	Secondary atom site location: difference Fourier map
*R*[*F*^2^ > 2σ(*F*^2^)] = 0.056	Hydrogen site location: inferred from neighbouring sites
*wR*(*F*^2^) = 0.140	H atoms treated by a mixture of independent and constrained refinement
*S* = 1.02	*w* = 1/[σ^2^(*F*_o_^2^) + (0.0491*P*)^2^ + 0.3356*P*] where *P* = (*F*_o_^2^ + 2*F*_c_^2^)/3
3155 reflections	(Δ/σ)_max_ = 0.001
214 parameters	Δρ_max_ = 0.14 e Å^−^^3^
1 restraint	Δρ_min_ = −0.19 e Å^−^^3^

### Special details

**Table:**

Geometry. All esds (except the esd in the dihedral angle between two l.s. planes) are estimated using the full covariance matrix. The cell esds are taken into account individually in the estimation of esds in distances, angles and torsion angles; correlations between esds in cell parameters are only used when they are defined by crystal symmetry. An approximate (isotropic) treatment of cell esds is used for estimating esds involving l.s. planes.
Refinement. Refinement of F^2^ against ALL reflections. The weighted R-factor wR and goodness of fit S are based on F^2^, conventional R-factors R are based on F, with F set to zero for negative F^2^. The threshold expression of F^2^ > 2sigma(F^2^) is used only for calculating R-factors(gt) etc. and is not relevant to the choice of reflections for refinement. R-factors based on F^2^ are statistically about twice as large as those based on F, and R- factors based on ALL data will be even larger.

### Fractional atomic coordinates and isotropic or equivalent isotropic displacement parameters (Å^2^)

**Table:**

	*x*	*y*	*z*	*U*_iso_*/*U*_eq_	
N1	0.0936 (3)	0.21111 (6)	0.6779 (2)	0.0459 (5)	
N2	0.2422 (3)	0.24250 (6)	0.7037 (3)	0.0472 (5)	
N3	0.2828 (5)	0.42054 (7)	0.6691 (3)	0.0671 (7)	
O1	−0.2996 (3)	0.18085 (5)	0.5833 (2)	0.0539 (5)	
H1	−0.2073	0.1987	0.6014	0.081*	
O2	−0.4045 (3)	0.04481 (5)	0.6539 (3)	0.0825 (7)	
O3	0.0352 (3)	0.28027 (5)	0.5016 (2)	0.0612 (5)	
O4	0.0861 (4)	0.42267 (6)	0.6147 (3)	0.0896 (7)	
O5	0.3980 (4)	0.44949 (6)	0.7108 (4)	0.1024 (8)	
C1	0.0134 (4)	0.14277 (7)	0.7182 (3)	0.0399 (5)	
C2	−0.2087 (4)	0.14565 (7)	0.6411 (3)	0.0415 (6)	
C3	−0.3392 (4)	0.11219 (7)	0.6220 (3)	0.0491 (6)	
H3	−0.4853	0.1143	0.5714	0.059*	
C4	−0.2565 (4)	0.07541 (7)	0.6771 (4)	0.0542 (7)	
C5	−0.0387 (4)	0.07155 (8)	0.7520 (4)	0.0576 (7)	
H5	0.0183	0.0468	0.7879	0.069*	
C6	0.0911 (4)	0.10532 (7)	0.7716 (3)	0.0484 (6)	
H6	0.2367	0.1029	0.8229	0.058*	
C7	0.1574 (4)	0.17699 (7)	0.7403 (3)	0.0429 (6)	
H7	0.2984	0.1743	0.8005	0.052*	
C8	0.1974 (4)	0.27655 (7)	0.6130 (3)	0.0426 (6)	
C9	0.3498 (4)	0.31068 (7)	0.6582 (3)	0.0402 (6)	
C10	0.2601 (4)	0.34854 (7)	0.6341 (3)	0.0450 (6)	
H10	0.1150	0.3519	0.5829	0.054*	
C11	0.3873 (4)	0.38098 (7)	0.6867 (3)	0.0485 (6)	
C12	0.6058 (4)	0.37744 (8)	0.7558 (3)	0.0571 (7)	
H12	0.6903	0.3999	0.7886	0.069*	
C13	0.6960 (4)	0.33984 (8)	0.7750 (3)	0.0555 (7)	
H13	0.8436	0.3369	0.8208	0.067*	
C14	0.5706 (4)	0.30664 (7)	0.7273 (3)	0.0471 (6)	
H14	0.6336	0.2814	0.7413	0.057*	
C15	−0.3370 (7)	0.00624 (8)	0.7178 (5)	0.1146 (14)	
H15A	−0.2171	−0.0031	0.6495	0.172*	
H15B	−0.4608	−0.0118	0.7015	0.172*	
H15C	−0.2877	0.0077	0.8454	0.172*	
H2	0.348 (3)	0.2395 (6)	0.796 (2)	0.051 (7)*	

### Atomic displacement parameters (Å^2^)

**Table:**

	*U*^11^	*U*^22^	*U*^33^	*U*^12^	*U*^13^	*U*^23^
N1	0.0437 (11)	0.0445 (12)	0.0478 (11)	−0.0099 (9)	−0.0071 (9)	0.0019 (9)
N2	0.0437 (12)	0.0440 (12)	0.0510 (12)	−0.0081 (9)	−0.0174 (10)	0.0054 (9)
N3	0.0820 (19)	0.0471 (15)	0.0735 (16)	−0.0082 (14)	0.0150 (14)	−0.0049 (12)
O1	0.0434 (10)	0.0495 (10)	0.0671 (11)	0.0005 (8)	−0.0078 (8)	0.0074 (9)
O2	0.0778 (15)	0.0504 (12)	0.1165 (18)	−0.0212 (10)	−0.0107 (12)	0.0072 (11)
O3	0.0612 (11)	0.0506 (10)	0.0663 (11)	−0.0004 (9)	−0.0333 (9)	0.0013 (9)
O4	0.0782 (16)	0.0575 (13)	0.132 (2)	0.0101 (11)	0.0040 (15)	−0.0009 (12)
O5	0.1152 (19)	0.0491 (12)	0.141 (2)	−0.0223 (13)	0.0003 (16)	−0.0134 (13)
C1	0.0397 (13)	0.0441 (14)	0.0359 (12)	−0.0019 (11)	0.0026 (10)	−0.0008 (10)
C2	0.0426 (14)	0.0422 (14)	0.0397 (12)	−0.0007 (11)	0.0028 (10)	0.0025 (10)
C3	0.0389 (14)	0.0531 (16)	0.0547 (15)	−0.0068 (12)	−0.0007 (11)	0.0025 (12)
C4	0.0579 (17)	0.0451 (15)	0.0595 (16)	−0.0141 (13)	0.0038 (13)	−0.0010 (12)
C5	0.0612 (18)	0.0455 (15)	0.0657 (17)	0.0033 (13)	0.0019 (14)	0.0053 (13)
C6	0.0414 (14)	0.0516 (15)	0.0518 (14)	0.0004 (12)	0.0008 (11)	0.0041 (12)
C7	0.0404 (13)	0.0491 (15)	0.0382 (12)	−0.0008 (11)	−0.0042 (10)	−0.0003 (11)
C8	0.0415 (13)	0.0453 (14)	0.0397 (12)	0.0023 (11)	−0.0055 (11)	−0.0004 (11)
C9	0.0390 (13)	0.0464 (14)	0.0345 (12)	−0.0073 (11)	−0.0015 (10)	−0.0002 (10)
C10	0.0434 (14)	0.0506 (15)	0.0409 (13)	−0.0033 (11)	0.0026 (11)	0.0033 (11)
C11	0.0557 (16)	0.0463 (15)	0.0447 (13)	−0.0066 (13)	0.0113 (12)	−0.0010 (11)
C12	0.0559 (17)	0.0647 (18)	0.0512 (15)	−0.0247 (14)	0.0071 (12)	−0.0048 (13)
C13	0.0415 (14)	0.0711 (19)	0.0534 (15)	−0.0141 (13)	−0.0004 (12)	0.0016 (13)
C14	0.0407 (14)	0.0562 (15)	0.0444 (13)	−0.0018 (11)	0.0034 (11)	0.0020 (11)
C15	0.138 (3)	0.0462 (19)	0.154 (3)	−0.029 (2)	−0.029 (3)	0.020 (2)

### Geometric parameters (Å, °)

**Table:**

N1—C7	1.281 (3)	C5—C6	1.378 (3)
N1—N2	1.386 (2)	C5—H5	0.9300
N2—C8	1.339 (3)	C6—H6	0.9300
N2—H2	0.900 (9)	C7—H7	0.9300
N3—O5	1.219 (3)	C8—C9	1.489 (3)
N3—O4	1.221 (3)	C9—C10	1.387 (3)
N3—C11	1.471 (3)	C9—C14	1.392 (3)
O1—C2	1.357 (2)	C10—C11	1.369 (3)
O1—H1	0.8200	C10—H10	0.9300
O2—C4	1.361 (3)	C11—C12	1.377 (3)
O2—C15	1.426 (3)	C12—C13	1.377 (3)
O3—C8	1.230 (2)	C12—H12	0.9300
C1—C6	1.387 (3)	C13—C14	1.376 (3)
C1—C2	1.414 (3)	C13—H13	0.9300
C1—C7	1.440 (3)	C14—H14	0.9300
C2—C3	1.372 (3)	C15—H15A	0.9600
C3—C4	1.380 (3)	C15—H15B	0.9600
C3—H3	0.9300	C15—H15C	0.9600
C4—C5	1.388 (3)		
			
C7—N1—N2	117.28 (18)	C1—C7—H7	119.6
C8—N2—N1	118.54 (17)	O3—C8—N2	122.4 (2)
C8—N2—H2	125.0 (14)	O3—C8—C9	120.9 (2)
N1—N2—H2	115.5 (14)	N2—C8—C9	116.69 (18)
O5—N3—O4	123.6 (3)	C10—C9—C14	119.1 (2)
O5—N3—C11	117.9 (3)	C10—C9—C8	116.77 (19)
O4—N3—C11	118.6 (2)	C14—C9—C8	124.1 (2)
C2—O1—H1	109.5	C11—C10—C9	119.3 (2)
C4—O2—C15	118.5 (2)	C11—C10—H10	120.3
C6—C1—C2	117.5 (2)	C9—C10—H10	120.3
C6—C1—C7	120.3 (2)	C10—C11—C12	122.1 (2)
C2—C1—C7	122.1 (2)	C10—C11—N3	117.9 (2)
O1—C2—C3	117.9 (2)	C12—C11—N3	120.0 (2)
O1—C2—C1	122.1 (2)	C13—C12—C11	118.3 (2)
C3—C2—C1	120.1 (2)	C13—C12—H12	120.8
C2—C3—C4	120.8 (2)	C11—C12—H12	120.8
C2—C3—H3	119.6	C14—C13—C12	120.8 (2)
C4—C3—H3	119.6	C14—C13—H13	119.6
O2—C4—C3	114.9 (2)	C12—C13—H13	119.6
O2—C4—C5	124.6 (2)	C13—C14—C9	120.2 (2)
C3—C4—C5	120.5 (2)	C13—C14—H14	119.9
C6—C5—C4	118.3 (2)	C9—C14—H14	119.9
C6—C5—H5	120.8	O2—C15—H15A	109.5
C4—C5—H5	120.8	O2—C15—H15B	109.5
C5—C6—C1	122.7 (2)	H15A—C15—H15B	109.5
C5—C6—H6	118.6	O2—C15—H15C	109.5
C1—C6—H6	118.6	H15A—C15—H15C	109.5
N1—C7—C1	120.8 (2)	H15B—C15—H15C	109.5
N1—C7—H7	119.6		

### Hydrogen-bond geometry (Å, °)

**Table:**

*D*—H···*A*	*D*—H	H···*A*	*D*···*A*	*D*—H···*A*
O1—H1···N1	0.82	1.90	2.618 (2)	146.
N2—H2···O3^i^	0.90 (1)	1.93 (1)	2.806 (2)	165 (2)

Symmetry codes: (i) *x*+1/2, −*y*+1/2, *z*+1/2.
